# Recurrent Pleural Effusion in an Elderly Patient With Chronic Myeloid Leukemia Following Tyrosine Kinase Inhibitor Therapy

**DOI:** 10.7759/cureus.85873

**Published:** 2025-06-12

**Authors:** Mohammad Abed Alhaleem, Muhammed Hussain, Ahmed Ahmed, Wafa Ahmed, Luxhman Gunaseelan, Andrew Easow

**Affiliations:** 1 Internal Medicine, Corewell Health Dearborn Hospital, Dearborn, USA; 2 Internal Medicine, Wayne State University, Detroit, USA; 3 Critical Care Medicine, Corewell Health, Dearborn, USA

**Keywords:** bilateral pleural effusion, chronic myeloid leukemia (cml), dasatinib, drug-related side effects and adverse reactions, imatinib, recurrent pleural effusion, tyrosine kinase inhibitor

## Abstract

Chronic myeloid leukemia (CML) is a myeloproliferative disorder treated with tyrosine kinase inhibitors (TKIs). While TKIs are effective in treating CML, their adverse effects can impact patient management. We present a case of an 83-year-old female diagnosed with CML in August 2024, initially treated with dasatinib but discontinued due to gastrointestinal toxicity. She was later started on imatinib in October 2024. One month later, she presented with progressive weakness and dyspnea, ultimately found to have a large pleural effusion requiring intervention. This case highlights the challenges in managing TKI-related adverse effects, particularly in elderly patients.

## Introduction

Chronic myeloid leukemia (CML) is a hematologic malignancy characterized by the presence of the BCR-ABL1 fusion gene, leading to uncontrolled proliferation of myeloid cells. The introduction of tyrosine kinase inhibitors (TKIs), such as imatinib, dasatinib, and nilotinib, has revolutionized the management of CML, transforming it from a fatal disease into a chronic, manageable condition [1]. TKIs work by inhibiting the activity of the BCR-ABL1 kinase, thereby suppressing malignant cell growth and promoting disease remission. However, despite their efficacy, TKIs are associated with a range of adverse effects, which can significantly impact patient adherence and quality of life [2].

Dasatinib, a second-generation TKI, is known for its higher potency but also its increased risk of adverse effects, including pleural effusion, pulmonary hypertension, and cytopenias. Pleural effusion in particular is a well-documented side effect, occurring in up to 35% of patients on dasatinib, and is often dose-dependent [3,4]. In contrast, imatinib, the first-generation TKI, has a lower risk of pleural effusions but can still lead to fluid retention, cardiotoxicity, and other systemic effects [5]. The management of these adverse effects in elderly patients is particularly challenging, as they often have multiple comorbidities that may exacerbate drug-related complications [6,7].

In this case, we present an elderly patient who developed recurrent pleural effusions following treatment with dasatinib and imatinib, emphasizing the need for careful monitoring and individualized treatment strategies.

## Case presentation

An 83-year-old female with a past medical history of hypertension, hyperlipidemia, and newly diagnosed CML started on dasatinib several days prior presented to the emergency department with severe fatigue, nausea, diarrhea, and a suspected gastrointestinal (GI) bleed. While admitted, she was found to have acute kidney injury (AKI), hypotension, and laboratory abnormalities, including a sodium level of 129 mEq/L, blood urea nitrogen (BUN) of 92 mg/dL, and creatinine of 11.64 mg/dL (Table 1). She required intensive care support with vasopressors, corticosteroids and intravenous fluids, with discontinuation of dasatinib. She eventually recovered and was discharged with close outpatient follow-up. Three months later, due to concerns about the progression of her CML, imatinib was initiated as an alternative treatment.

**Table 1:** Labaratory Abnormalities

Test	Lab Value	Reference Range
Serum Sodium (Na)	129 mEq/L	135 - 145 mEq/L
Serum Creatinine (Cr)	11.64 mg/dL	0.50 - 1.10 mg/dL
Blood urea nitrogen (BUN)	92 mg/dL	7 - 25 mg/dL

**Figure 1:**
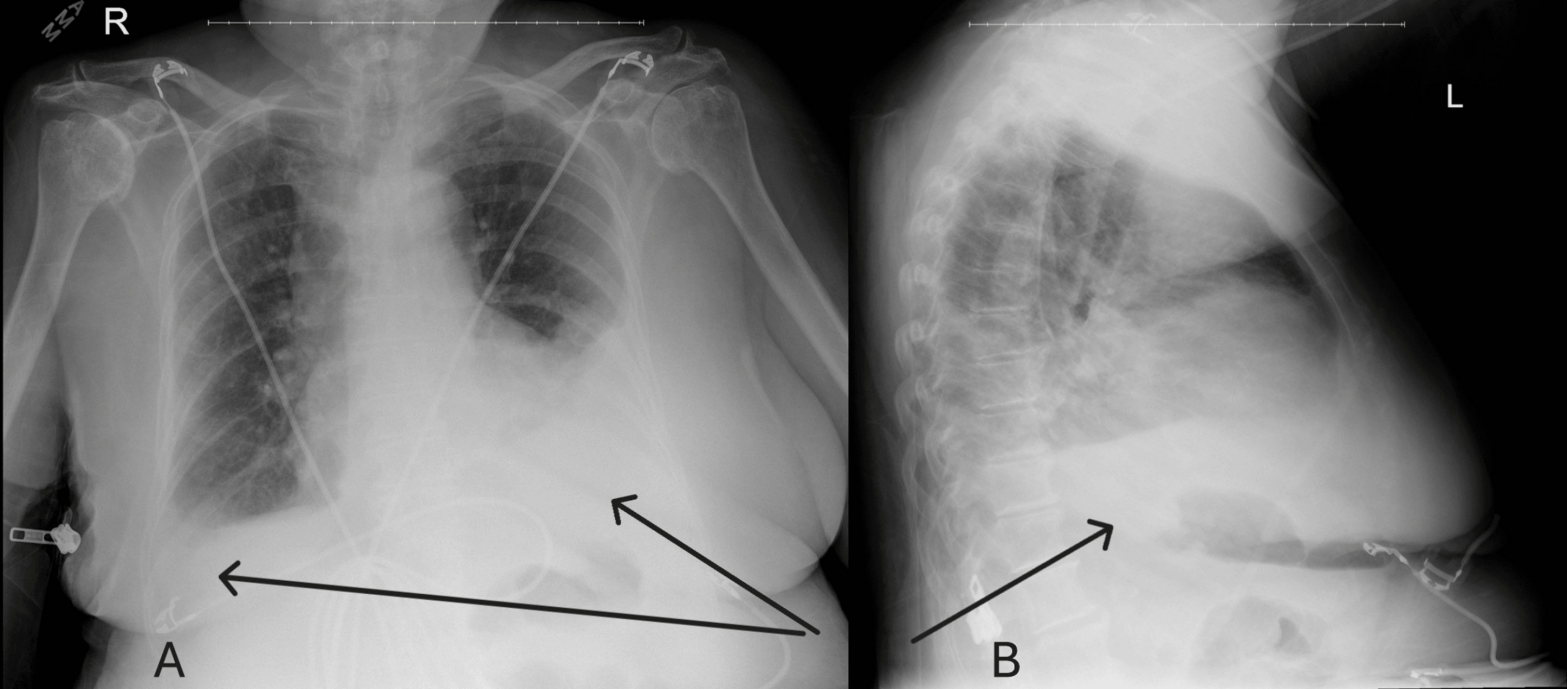
A: Anterior-Posterior Chest X-Ray. B: Lateral Chest X-Ray. Note the bilateral pleural effusions.

**Figure 2:**
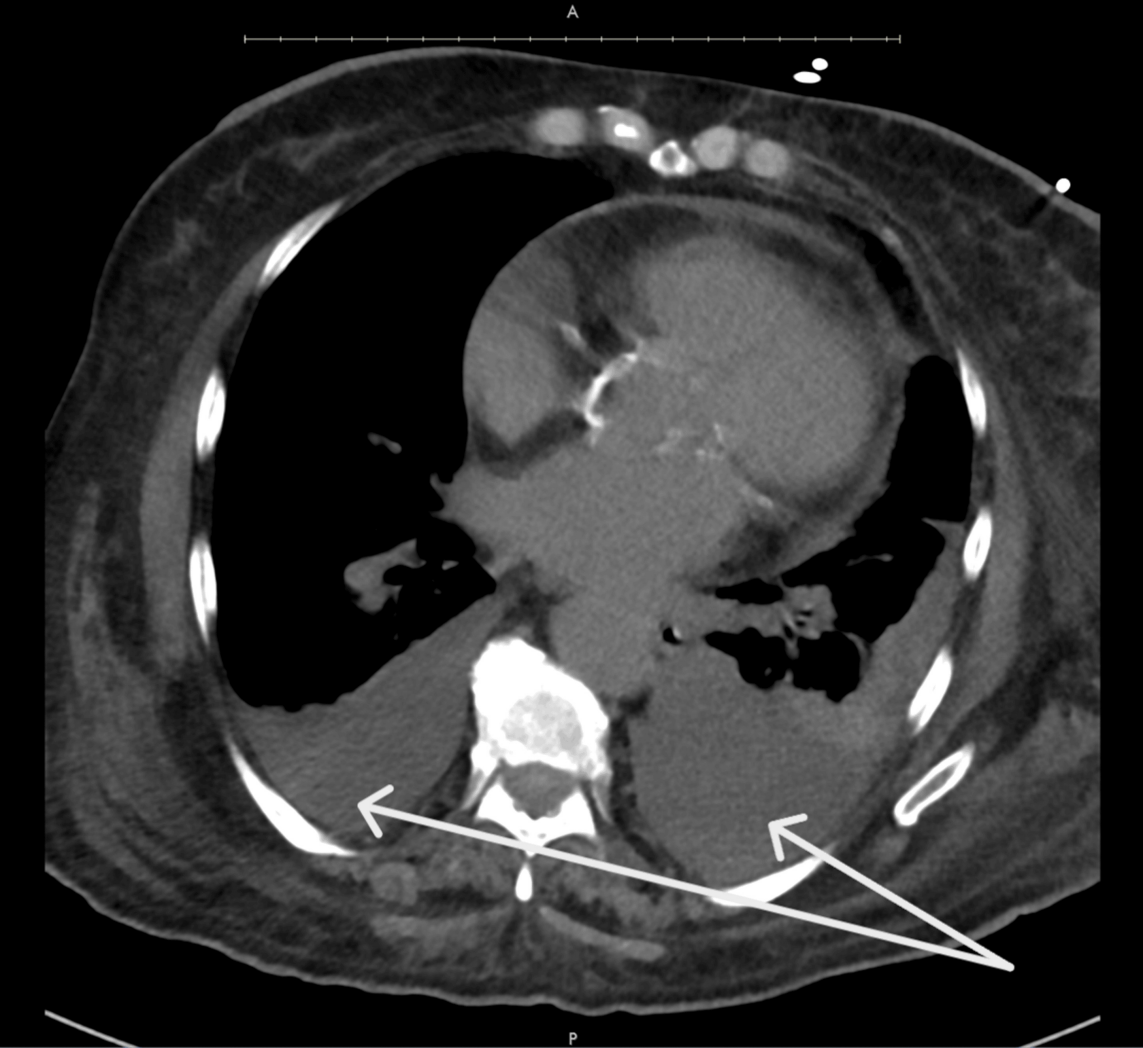
CT Chest. Note the Moderate Bilateral Effusions With Atelectasis and Small Pericardial Effusion.

However, one month later, the patient presented again to the emergency department with worsening generalized weakness and new-onset shortness of breath over the past two weeks. She denied fever, chills, or cough. On admission, oxygen saturation was normal. A chest X-ray revealed a moderate left-sided pleural effusion and a smaller right-sided effusion (Figure 1), which was re-demonstrated on a follow-up CT chest (Figure 2). 

Notably, a CT scan performed during her previous admission four months ago had not shown pleural effusions, suggesting this was a new development. Laboratory workup showed a hemoglobin level of 9.5 g/dL with a normal platelet count. A venous Doppler ultrasound of the left upper extremity did not reveal deep vein thrombosis (DVT). Pulmonology was consulted for further evaluation of the effusions.

The patient underwent thoracentesis, with approximately 600 mL of fluid removed. She was subsequently started on diuretics. A transthoracic echocardiogram (TTE) revealed a preserved ejection fraction but demonstrated mitral stenosis (MS) and mitral regurgitation (MR). A transesophageal echocardiogram (TEE) confirmed mild-to-moderate MR and moderate MS. Given her cardiac findings, a conservative approach with medical management was pursued.

The patient was discharged on diuretics with close outpatient follow-up with cardiology, pulmonology, and hematology. Given her recurrent pleural effusion and intolerance to dasatinib and imatinib, further discussion regarding CML treatment options was planned on an outpatient basis.

## Discussion

Pleural effusion is a well-recognized adverse effect of TKIs, particularly dasatinib, which is thought to result from increased vascular permeability and immune-mediated mechanisms [3,8]. The exact pathophysiology remains unclear, but potential mechanisms include endothelial dysfunction, inhibition of platelet-derived growth factor receptor (PDGFR), and immune cell activation leading to an inflammatory response [4,8]. In some cases, dose reduction or temporary discontinuation of the drug may resolve the effusion, but recurrent or severe cases often necessitate switching to an alternative TKI [4,9].

Although dasatinib is the most frequently implicated TKI in pleural effusion, imatinib has also been associated with fluid retention and, in rare cases, pleural effusion [5]. The risk is higher in elderly patients and those with underlying cardiac disease, as seen in this patient. Risk factors for TKI-induced pleural effusion include advanced age, preexisting cardiovascular disease, and prior TKI intolerance, all of which our patient had [6,7,10].

Management strategies for TKI-induced pleural effusions range from conservative measures such as diuretics and steroids to invasive interventions like thoracentesis in severe cases. In patients with recurrent pleural effusions, alternative therapies such as bosutinib or reduced-dose nilotinib may be considered, as these agents have lower rates of pleural effusion [2,7]. However, switching therapies requires careful consideration of efficacy, side effect profiles, and patient preference. The decision to transition to an alternative TKI should take into account the patient’s comorbidities, tolerance to previous treatments, and the likelihood of disease control with the new agent. Certain TKIs, such as bosutinib, have been associated with a lower incidence of pleural effusions and may be preferable in patients with recurrent fluid retention. However, bosutinib is associated with gastrointestinal disturbances and hepatotoxicity. Invariably, some patients may prefer to discontinue therapy due to the cumulative burden of side effects, emphasizing the importance of shared decision-making between the clinician and patient [6,10]. Alternative dosing strategies, such as reducing the dose of the current TKI or implementing a drug holiday, may also be considered in select cases to minimize adverse effects while maintaining therapeutic efficacy.

## Conclusions

This case illustrates the potential for recurrent pleural effusions in elderly patients with CML receiving TKIs, even when switching between agents. It emphasizes the need for vigilant monitoring, early intervention, and individualized treatment strategies to optimize patient outcomes. Given the high risk of pleural effusion with dasatinib and, to a lesser extent, imatinib, clinicians should consider predisposing risk factors before initiating therapy. Future research is needed to identify predictive markers for TKI-induced pleural effusions and to develop alternative treatment strategies for high-risk patients.
